# Understanding the research capacity and culture of Australian podiatrists

**DOI:** 10.1186/1757-1146-8-S2-O42

**Published:** 2015-09-22

**Authors:** Cylie Williams, Peter Lazzarini

**Affiliations:** 1 Peninsula Health, Community Health, Frankston, VIC, 3199, Australia; 2 Monash University, Department of Physiotherapy, VIC, 3199, Australia; 3 School of Clinical Sciences, Queensland University of Technology, Brisbane, Queensland, 4059, Australia; 4 Allied Health Research Collaborative, Metro North Hospital & Health Service, Queensland Health, Rode Rd, Brisbane, Queensland, 4032, Australia

**Keywords:** research, culture, capacity, Australia, podiatry

## Background

Enhancing evidence based practice via the rapid translation of new research evidence into every day clinical practice is fundamental to the success of health care and in turn health care professions. There is little known about the research capacity and culture of the podiatry profession across Australia. This study aimed to investigate the research capacity and culture of the podiatry profession within Australia and determine if any factors influenced skills and success.

## Methods

All Australian podiatrists were eligible to participate in a cross-sectional online survey. Podiatry Associations disseminated the survey and podiatrists encouraged to distribute it to colleagues. The Research Capacity and Culture (RCC) tool was used to collect research capacity and culture item variables using a 10-point scale (1=lowest; 10=highest). Additional demographic, health sector, workplace data variables were also collected with quantitative and qualitative questions about enablers and barriers to participating in research projects and translating evidence.

## Results

There were 229 fully completed surveys (6% of Australian registered podiatrists). Respondents reported low skill levels (Median < 4) on the majority of individual research skill items, except for locating and critiquing research literature. More recently graduated, male podiatrists working in multi-practitioner workplaces reported higher individual skill and success to undertake early phase research activities (p<0.05). Higher individual skill and success in the later phases of research projects such as analysis of data, writing for publication were only associated with working in a multi-practitioner workplace. Non-clinical and public health sector podiatrists reported significantly higher post-graduate study involvement, research activity involvement, provisions to undertake research and individual research success and skill than those working in the private health sector (*p* < 0.05).

## Conclusions

Podiatrists in Australia had similar low levels of research skill levels to those reported in other allied health professions. Interestingly, the workplace setting, gender and recency of practice is associated with some of the research skills in individuals. This is important knowledge for podiatrists and researchers aiming to translate research evidence into clinical practice.

